# Case report: Psychotherapy for enhancing psychological adjustment to dysphoric milk ejection reflex

**DOI:** 10.3389/fpsyt.2024.1333572

**Published:** 2024-01-31

**Authors:** Reem Deif

**Affiliations:** Institute of Global Health and Human Ecology, School of Sciences and Engineering, American University in Cairo, New Cairo, Egypt

**Keywords:** postpartum mental health, dysphoric milk ejection reflex, body shaming, psychotherapy, breastfeeding

## Abstract

Given its endless benefits, breastfeeding is widely acknowledged as the optimal choice for both maternal and infant health. Nevertheless, breastfeeding mothers often encounter various challenges that may hinder their ability to fully embrace this experience. This report delves into a compelling case of Dysphoric Milk Ejection Reflex (D-MER), a largely underexamined mental health issue among lactating mothers. D-MER is characterized by intense aversion right before milk let down, which can significantly impede a mother’s willingness to breastfeed. The primary aim of this case report is to provide a comprehensive psychological understanding of D-MER, emphasizing aspects of attachment, the transition into motherhood, and the sociocultural sexualization of the female body. We also offer an overview of the psychotherapeutic journey, highlighting key insights and progress achieved over a span of six months. Therapy adopted an integrative approach combining narrative techniques and skills training such as mindfulness to facilitate a comprehensive therapeutic experience. This case underscores the psychological dimensions of the breastfeeding experience, complementing the well-established biochemical and physiological aspects of D-MER. It also emphasizes the need for further research into the psychological facets of both successful and less successful breastfeeding experiences.

## 1 Introduction

Dysphoric milk-ejection reflex (D-MER) refers to the mother’s feeling of aversion right before milk let down characterized by intense dysphoria which may eventually interfere with the mother’s ability to breastfeed regularly (1). Physical sensations, such as “a hollowness in the pit of the stomach”, might be experienced, but other emotional symptoms might include helplessness, hopelessness, guilt, worthlessness and “a desire to hide from the world” that are only experienced around the milk release. Although this dysphoria disappears after milk let-down, usually within 90 to 120 seconds, the recurrence with every breastfeeding session increases its psychological burden. Between each D-MER, a lactating mother can experience regular emotions including happiness and feeling connected to the infant once again. It is also important to note that D-MER is not only triggered by direct nipple stimulation, but also, the release of milk in response to thinking about the baby, pumping or milk expression, breast fullness, etc. With each episode, a mother may also experience major difficulties with concentration (2). The only statistics available estimate the prevalence of D-MER to be 9.1% (3), being on average in the same range as postpartum depression which has an estimated prevalence of 6.5% to 20% as per the latest update of the National Library of Medicine (4). This is ironic because, to the best of our knowledge, a total of five cases of D-MER have been published (see 2, 5–7). In the absence of published cases involving Arab mothers with D-MER, the primary aim of this case report is to provide a comprehensive psychological understanding of D-MER in a psychosocial context, emphasizing aspects of attachment, the transition into motherhood, and the sociocultural sexualization of the female body. It also provides important insights about the clinical aspects of D-MER and how they have been addressed therapeutically.

One condition that might initially resemble D-MER is breastfeeding aversion/agitation (BAA). While both involve negative emotional experiences, in BAA mothers have the impulse of detaching the latching infant due to the continuation of the aversive feelings throughout the lactation session, unlike D-MER where dysphoric feelings only arise before milk letdown and then dissipate. Another core difference is the nature of the emotions experienced in D-MER there is a feeling of dread, dysphoria, or sadness unlike BAA which is primarily marked by anger and agitation (8).

D-MER is not regarded as a psychological disorder, but rather a physiological one primarily influenced by hormonal factors (7). To begin with, breastfeeding involves increased production of oxytocin that also affects how quickly cortisol levels drop during breastfeeding. Higher levels of circulating oxytocin are associated with greater activation of the brain’s mesocorticolimbic reward system (9). Therefore, a decrease in oxytocin levels might contribute to the unpleasant symptoms experienced in D-MER. Another theory postulates the role of dopamine in D-MER based on the finding that dopamine reuptake inhibitors play a role in eliminating D-MER symptoms (2).

While there are still currently no approved treatments for D-MER, various recommendations for managing its symptoms have been suggested such as skin-to-skin contact with the infant, meditation and relaxation practices, and behavioral distractions (10). Additionally, given its neurobiological basis, there is an emerging hope that dopamine reuptake inhibitors such as bupropion could possibly help in alleviating D-MER symptoms as per one case report. Pseudoephedrine, known for its ability to halt milk production, also effectively eradicated D-MER not by influencing oxytocin, but through the reduction of prolactin levels. However, it is still unclear if this effect is due to a direct increase in dopamine levels (2).

## 2 Case description

### 2.1 Patient information

MS is an Egyptian 32-year-old female. She works as a software developer, but is currently on maternity leave. She has been married for 3 years and has two boys aged 2.5 years and 2 months. She presented with a primary complaint of intense postpartum mood disturbances that she only experienced the first 60-90 seconds of each lactation session, starting only with milk let down, and which recurred with each milk ejection in every feeding session. The emotional response is usually brief, yet intense, and ends soon after the milk ejection. She described it as an “intense, hollow sensation in her chest and upper stomach as if falling off a roller coaster” that is usually accompanied with self-hate thoughts, a desire to escape from the moment and low self-esteem. In an attempt to reduce the frequency of such “attacks”, she indirectly lactates by using an electric pump and then bottle-feeds her newborn. While this approach does not diminish the intensity of her emotional experience, it leads to a greater volume of expressed breast milk. This increase permits her to forego several milk expression sessions, in contrast to direct lactation, which necessitates her availability every 2-3 hours or as needed. MS feels torn between her desire to escape from lactation and her guilt feelings for not wanting to do it. She described herself as an anxious mother who is constantly driven by her sense of uneasiness.

MS reported symptoms such as crying, inattention, forgetfulness, and social withdrawal following the birth of her second child besides an ongoing sense of anticipation. Her symptoms often translated to somatic symptoms of anxiety (i.e.: bowel problems, tension headaches, and heart palpitations). By taking history, it became clear that her current clinical profile was typical to her profile following the birth of her first child two years earlier with a period of remission in between. It was also evident that some of her depressive symptoms were secondary to her anxiety.

In terms of current functioning, MS experiences an average of 5 hours of interrupted sleep daily as her newborn is still in the process of establishing a consistent sleep routine. She reported good appetite and occasional instances of emotional eating. In terms of coping, she chose a strategy of social withdrawal from family and friends to evade potential triggers that often resulted in emotional dysregulation. One major trigger is the recollection of her episiotomy, which she considered as a violation of her body. Other triggers included criticisms about the way she handles her children and hearing about conflicts between other family members. However, her husband has been emotionally present and supportive and is the only one she chooses not to distance herself from. MS identified her main strength as being a hard-worker, yet reflected on her self-doubts of not being good enough, especially when it comes to being a mother.

MS has been self-referred to therapy after she “forgot her newborn in her car a week earlier”. She consulted a psychiatrist who was not familiar with her “breastfeeding condition” and suggested escitalopram, a selective serotonin reuptake inhibitor having both antidepressant and anxiolytic effects, for trial which she did not agree to take because she did not want it to pass onto her baby through breastmilk.

### 2.2 Medical history

MS also has a long history of hypoglycemia and hypotension that was first identified during her middle/late childhood. As a newly-wed, she experienced vaginismus that was only resolved following an episiotomy in her second delivery. She also had one unexplained threatened abortion the second trimester of her second pregnancy.

Being overdue in both her pregnancies, she received medical induction. In the first delivery, only a membrane sweep and the administration of prostaglandin were needed to induce labor compared to her second birth which required amniotomy and the administration of Pitocin in addition to prostaglandin and a membrane sweep. Although both deliveries were vaginal, the fear of having a c-section was always haunting her. She experienced panic attacks or “epidural anxiety”, following the administration of an epidural in both deliveries triggered by numbness sensations.

Based on the most recent feedback from her breastfeeding consultant, MS had abnormal nipples that required special nipple shields for the baby to latch. Although she achieved success with the use of nipple shields several times, she ultimately decided against direct breastfeeding because of emotional aversion. This led her to occasionally miss some lactation sessions or at least delaying them. As a result, she had some secondary complications including breast engorgement leading to second-degree lactational breast mastitis two weeks earlier.

### 2.3 Psychiatric history

MS reported a positive family history of anxiety; her mother is diagnosed with generalized anxiety disorder and is currently on psychopharmacological treatment. MS herself was diagnosed with generalized anxiety disorder five years ago which was also the time when she had a comorbid depressive episode secondary to her anxiety. She was in therapy and pharmacological treatment for 18 months followed by a remission for the past 4 years. She was initially prescribed mirtazapine, venlafaxine and finally vortioxetine. No high-risk behaviors or suicidal ideations have been identified.

### 2.4 Psychosocial history

MS comes from an upper-middle-class family in Cairo. She is the second child of an “anxious” mother and a “workaholic” father. Her sister is 5 years older, married, and lives in Saudi Arabia. MS mentioned that she was not breastfed by her mother because her mother “was too shy to do it”. MS had exceptional school performance, has been successful in her career and could maintain long-term relationships despite her anxiety. She feels pride in the fact that “she chose her husband” when they first met and that it was not a typical arranged marriage like the majority of girls in her community. No history of abuse was reported.

MS identifies as a practicing Muslim who finds “comfort in prayers and in talking with God”. She added that she experienced a sense of pride in being divinely chosen to be blessed with her children, yet concurrently, she struggled with feelings of guilt over her perceived inadequacy as a mother.

## 3 Diagnostic assessment

Besides D-MER, MS met criteria for generalized anxiety disorder (GAD) as per the Diagnostic and Statistical Manual for Psychiatric Disorders-5 (11).

### 3.1 Formulation

This case formulation is grounded in the psychodynamic framework with an emphases on psychological mindedness, and the exploration of internal conflicts that are possibly shaped by past experiences.

MS displayed good psychological mindedness which has been facilitating her progress in therapy. The therapist has helped MS reach her own formulation of her problem which was “my body has failed me”. She also reflected on how her body has only been serving others over the past 4 years; namely, her husband and children, leaving her torn between her conflicting obligations as a wife and a mother.

Looking into the history of her experience with her body, MS mentioned instances of “her body unexpectedly failing her” including her feeling physically vulnerable as a child with hypoglycemia and hypotension. She also doubted her femininity after trying to manage her vaginismus without medical intervention and failing at this. This sense of self-doubt was reinforced by her body’s inability to be “naturally induced for labor”, the negative breastfeeding experiences she had with both babies, the occasional diminished milk supply, and the threatened abortion she experienced in her second pregnancy. Based on such experiences, MS “did not trust her body” to produce enough milk for her newborn which added more distress to her D-MER condition making breastfeeding more “aversive”. Following up on this, the themes below emerged in therapy and were directly linked to D-MER:

A. The mother-infant attachment and the motherhood experience

The experience of cognitive-emotional dissonance between “I must breastfeed to be a good mother” and the dysphoria MS feels as an intense embodied sensation with every lactation session can itself impact not only her perception of her image as a mother, and therefore her acceptance, but also the way she relates to the newborn. Similar to her newborn’s experience of Melanie Klein’s paranoid-schizoid position, MS projects her feelings on herself as an external object and then internalizes this as part of her self-concept. Only when she perceives herself as a nurturing mother who is able to follow a strict lactation schedule, she internalizes the “good” part of herself. Dealing with a great deal of anxiety and mother guilt, she is judging herself as a bad mother and she has difficulty integrating the good and bad parts of herself. MS ‘s low sense of self-efficacy when it comes to breastfeeding is a major perpetuating factor of her symptoms. Her feelings of incompetence, especially in comparison to other mothers who are not only able to breastfeed, but who also enjoy breastfeeding, threaten her sense of maternal autonomy. In other words, she has failed to have a personal meaning of breastfeeding other than an obligation, in addition to lacking a model of breastfeeding in her own mother. Although she did not feel abandoned by her mother for this reason, she has difficulties today embracing her mother identity.

B. The sexualization of the female body

MS lacks a sense of body ownership believing that her body has been sexualized as if it was not hers. She recalled her mother nagging her to wear teeth braces to look good, her mother-in-law’s comments on her need to exercise to lose her “mama belly”, her husband’s concerns about her vaginismus, her gynecologist’s decision to do a “husband stitch” in her first delivery to be corrected in an episiotomy in her second delivery without her consent, pressure by the nurses to breastfeed in the hospital room while family members were visiting, and finally criticism of her decision to indirectly lactate using a pump which, according to some family members, might not help her bond with her newborn and which only triggered her guilt feelings.

Anxiety about bodily exposure is another perpetuating factor that has been reinforced throughout her upbringing which valued conservativeness and modesty. This started during her adolescence with comments from teachers on her dresses not being conservative enough. More specifically, the sexualization of a woman’s breasts besides the need to expose them to breastfeed, depending on the level of privacy provided, might have created a sense of confusion for MS as a nursing mother who is not used to being physically exposed.

### 3.2 Therapy structure over the past 6 months

The therapist followed an integrative approach with narrative and psychodynamic components to help the client verbalize her thoughts and emotions, increase her awareness of their roots, and develop positive narratives focused on her experience with her body (i.e.: giving birth, physical health, breastfeeding, body scars, etc.) and her sense of body ownership through a corrective emotional experience.

Additionally, the themes outlined in the case formulation were collaboratively developed and processed with the client during the therapy sessions. This narrative approach underscores the significance of fostering self-agency in the client, emphasizing its vital role in making the therapeutic process effective. This is especially effective with clients in the Egyptian culture who might exhibit a reluctance to ask for help and demonstrate a preference for independently solving their issues.

Specific techniques included the following:

A. Processing her full range of emotions both in the past and the here-and-now, where they are coming from, how they have shaped her experiences, and how she can reexperience them more therapeutically in the here-and-now. This involved a combination of psychodynamic techniques including free association besides working in the here-and-now and doing bodily focusing.
B. Writing her labor diaries to retell her birthing experience in a safe therapeutic setting to build up her emotional resilience towards this event. From a narrative perspective, the therapist helped by questioning and reflecting to help MS “re-author her mother identity”.
C. Practicing basic mindfulness skills on eating, bathing, and breathing to re-befriend her body and reinforcing insights gained through mindfulness in daily journaling.
D. Practicing assertiveness skills to help her overcome the anxiety that leads her to withdraw and therefore reduce instances of avoidance. A special focus has been put on encounters where someone commented on her breastfeeding, body, mother role, etc.

MS attended a total of 25 therapy sessions on a weekly basis over a period of six months. She and the therapist collaboratively decided on the termination of therapy, with a plan to conduct monthly check-ins for ongoing assessment and support with the same therapist, especially for her GAD symptoms. Although she still experiences D-MER, dysphoria is significantly less in terms of duration and intensity. Rather than “externalizing her breastfeeding problem”, MS is now able to “own her breastfeeding condition” and feel a sense of pride that she has been able to overcome such a challenge and play the mother role the way she “chose to” (i.e.: indirect lactation instead of direct breastfeeding). In other words, she has reached a point of acceptance regarding her body’s limitations, acknowledging that indirect lactation is the most feasible option for her and that she needs to honor her body. This has given her a sense of empowerment that she is still working on in therapy. Apart from breastfeeding, MS reflected on her ability to be more mindful of her encounters with her children, besides being more comfortable in her body. Focusing on the process of therapy, she stated that “she is not there yet” referring to her symptoms of anxiety which she is still learning to navigate along other emotions. Interestingly, her anxiety is now mainly triggered and situational rather than generalized. MS ‘s husband also shared that she is more proactive in different interpersonal encounters and that she does not withdraw as before.

## 4 Discussion

This paper presented a case study of D-MER highlighting its distinct nature from other breastfeeding-related conditions such as BAA. The specificity of D-MER lies in the timing and nature of the negative emotional experiences, which are closely tied to the moments of milk let-down. Understanding such differences is crucial for the accurate diagnosis and the provision of appropriate support for breastfeeding mothers with this condition. To understand the psychopathology of D-MER, motherhood should be conceptualized from a psychological perspective as an independent developmental stage in a woman’s lifespan. It can significantly exacerbate the emotional distress experienced by new mothers, who are also grappling with various new challenges, such as changes in relationships, concerns about their marital relationships, health issues, and career adjustments, among many other challenges (12). Additionally, the patient’s Middle Eastern background contributes to the complexity of this case, as it places importance on issues related to body image, femininity, and sexualization. Put together with her earlier problem with vaginismus, it might be argued that MS experiences Johnston-Robledo and colleagues’ (13) concept of “reproductive shame” coming from the objectification of her female body. According to their findings, as a health behavior, women with higher levels of body shame also have shameful attitudes towards reproductive functions, including breastfeeding.

The central message of this case report underscores that while D-MER may be a physiological one primarily influenced by hormonal factors (7), the application of psychotherapy can assist patients in achieving a sense of “acceptance.” This acceptance can help minimize the interference of D-MER symptoms with their overall well-being and daily functioning. The efficacy of psychotherapy in achieving these outcomes stems not just from its capacity to foster self-regulation skills and promote lifestyle modifications, which, as noted by Liu and colleagues (7), can offer relief to breastfeeding mothers dealing with D-MER symptoms. It also lies in its deep processing of emotional experiences and the management of other psychological issues that may overlap with D-MER such as symptoms of postpartum depression and postpartum anxiety as in the case of MS. In light of this, providing tangible tools to support mothers in addressing breastfeeding challenges may prove more effective than merely raising awareness about the advantages of breastfeeding and inducing guilt for not being able to breastfeed.

While this case report offers insights into D-MER, we are still aware of its limitations in terms of generalizability for different reasons. First, the experiences of those dealing with D-MER may vary widely from one person to the other. The therapeutic intervention used here might not be equally effective with others in different cultures, so cross-cultural validity is not determined. Additionally, there is room for self-selection bias given that MS has self-referred to therapy which might reflect some level of readiness for change that is not necessarily the case with other women with D-MER. This is in addition to the lack of objective progress measures, given the nature of psychotherapy, making it primarily based on self-reports and therapist observations. The client also showed a negative attitude towards using such assessments. Such resistance is frequently observed in cultural contexts like Egypt, where clients often prioritize the need to be “heard” in therapy (as shown in the patient’s perspective section) sessions and express reluctance towards standardization and quantification of therapy outcomes. Consequently, the choice to refrain from using psychometric assessments was made primarily to foster a strong therapeutic rapport and to demonstrate cultural sensitivity. Finally, the duration of therapy might not guarantee the long-term effects and their sustainability in the future, potentially with future breastfeeding experiences with new babies. In this regard, a more extended follow-up with a larger sample size would be helpful and this case study, although not completely generalizable, might inspire other studies with stronger merits.

## 5 Patient perspective

I’ve witnessed my personal growth during my therapy journey. While I’ve been in therapy before, this time has been the most profound. Initially, I had doubts not only about the effectiveness of therapy and my therapist, but also about opening up because I had a deep-seated belief that nothing would really change, so why bother? But now, I’ve reached a point where I can view my body from a different perspective. I’ve learned to appreciate my femininity and the privilege of being a “biological” mother with all that it entails; the good and the bad, the easy and the difficult, the spoken and the unspoken. Looking back at the past few months, there were definitely moments when therapy felt incredibly painful, and I would end my sessions in tears, not wanting to return the following week. I can vividly recall times when my heart would race just before my therapist showed up for the session. It was as if my body was resisting the change, not being ready yet for healing. However, my therapist patiently gave me all the time I needed, and I’m grateful for this.

Throughout this journey, my therapist consistently demonstrated acceptance and support. When I first began therapy, I felt empty, but with each time she genuinely listened to me, I began to feel more complete and emotionally contained. While I initially thought I was dealing with postpartum depression, or potentially anxiety, I can now confidently say that such a label doesn’t fully capture the complex experience that we, as proud mothers, go through. Breastfeeding is difficult, motherhood is difficult, childbearing is difficult. Yes, there must be some biological basis for such “difficulties”, but what helps us the most is to be heard and supported throughout this journey.

## 6 Conclusion

In conclusion, this case report on D-MER highlights the intricate interplay between biopsychosocial processes in the context of breastfeeding. The story of MS does not only highlight the challenges associated with this condition, but also the potential of psychotherapy in supporting mothers with D-MER. While still primarily viewed as a physiological condition, the application of psychotherapy demonstrates its significant role in fostering acceptance, as a therapeutic factor, and minimizing the impact of D-MER symptoms on the overall wellbeing. This case also suggests the importance of contextualizing breastfeeding as a cultural practice influenced by norms, beliefs and social expectation. This case, while not necessarily representative of others, offers a valuable perspective that can potentially inform future research on D-MER in order to provide better support to other mothers.

## Data availability statement

The raw data supporting the conclusions of this article will be made available by the authors, without undue reservation.

## Ethics statement

The studies involving humans were approved by Prof. Mona Yehia Rakhawy, Department of Psychiatry, Kasr Al Ainy, Cairo University School of Medicine. The studies were conducted in accordance with the local legislation and institutional requirements. The participants provided their written informed consent to participate in this study. Written informed consent was obtained from the individual(s) for the publication of any potentially identifiable images or data included in this article.

## Author contributions

RD: Conceptualization, Writing – original draft, Writing – review & editing.
